# What Makes a Good Home-Based Nocturnal Seizure Detector? A Value Sensitive Design

**DOI:** 10.1371/journal.pone.0121446

**Published:** 2015-04-13

**Authors:** Judith van Andel, Frans Leijten, Hans van Delden, Ghislaine van Thiel

**Affiliations:** 1 University Medical Centre Utrecht, Department of Clinical Neurophysiology, Utrecht, The Netherlands; 2 University Medical Centre Utrecht, Julius Center for Health Sciences and Primary Care, Utrecht, The Netherlands; Illinois Institute of Technology, UNITED STATES

## Abstract

A device for the in-home detection of nocturnal seizures is currently being developed in the Netherlands, to improve care for patients with severe epilepsy. It is recognized that the design of medical technology is not value neutral: perspectives of users and developers are influential in design, and design choices influence these perspectives. However, during development processes, these influences are generally ignored and value-related choices remain implicit and poorly argued for. In the development process of the seizure detector we aimed to take values of all stakeholders into consideration. Therefore, we performed a parallel ethics study, using “value sensitive design.” Analysis of stakeholder communication (in meetings and e-mail messages) identified five important values, namely, health, trust, autonomy, accessibility, and reliability. Stakeholders were then asked to give feedback on the choice of these values and how they should be interpreted. In a next step, the values were related to design choices relevant for the device, and then the consequences (risks and benefits) of these choices were investigated. Currently the process of design and testing of the device is still ongoing. The device will be validated in a trial in which the identified consequences of design choices are measured as secondary endpoints. Value sensitive design methodology is feasible for the development of new medical technology and can help designers substantiate the choices in their design.

## Introduction and Aim

Epilepsy is one of the most common chronic neurological disorders and affects 0.5% of the Western population [1]. Despite a rapid increase in pharmaceutical and surgical treatment options, 30% of epilepsy patients have intractable seizures and are faced with the prospect of having to cope with them for a prolonged time, even their entire life.

In an unsupervised environment, seizures can be dangerous (falls, injuries as a result of violent movements, confusional wandering) and may evolve into status epilepticus. For this reason, the reliable detection of seizures in an early stage might facilitate timely and adequate care. This is particularly true for nocturnal seizures, when patient supervision is difficult. Indeed, seizures occurring during sleep often go unnoticed, especially in patients who sleep alone, and seizure-related sudden death occurs most often during the night [2]. However, the detection of nocturnal seizures by means of a medical device is challenging, because movements during sleep may trigger a false alarm, with both patient and caregiver being woken unnecessarily.

In a collaborative project, the University Medical Centre Utrecht and two Dutch epilepsy centers (SEIN and Kempenhaeghe) formed the TeleEpilepsy consortium in 2010, with a view to developing a multimodal device to detect nocturnal seizures. The detector uses information from a video camera, microphone, movement sensors, and ECG to detect epileptic seizures at night and alerts the patient’s caregiver. It is primarily intended for use in children younger than 16 years and mentally impaired adolescents and adults with major nocturnal seizures.

### Value Sensitive Design

Values are general beliefs about preferred behavior, states, or courses of action. Values reflect a person’s or a group’s ideas about how things ‘ought to be’. The design of medical technology is not value neutral: perspectives of users and developers are influential in design, and design choices influence these perspectives [3]. Research shows that different stakeholders can have different value systems and different conceptual frameworks. For example, Zwart et al. [4] showed that researchers and users of a new wastewater treatment technology had different ideas about what constituted ‘risk’ and about responsibility for undesired side effects.

During the development of new medical technologies, the interaction between stakeholder values and design choices is generally ignored. To investigate the possible interaction between design choices and stakeholder values during the development of the seizure detector, a so-called *parallel ethics study* was performed to stimulate discussion of values that might interact with design choices. A method to make values, value tensions, and value trade-offs in technology design explicit is Value Sensitive Design (VSD)[5]. VSD emerged from insights gained from studies of human-computer interactions in the 1990s [6] and provides a framework for ethical considerations in technological design.

### Aim

We aim to do two things: first, we describe stakeholders and their moral values that are relevant to the development and use of the seizure detector. Second, we present four design choices and the corresponding risks and benefits in terms of the identified values. The values and design choices can inform the decision making about further development. The seizure detector is currently being investigated in a clinical setting, to compare its performance with that of the gold standard of in-hospital EEG-video registration. Thereafter, the device will be optimized for use in institutionalized or home-based care.

## Method

A professional ethicist (GT) was involved from the start of the design process. The other researchers involved were a medical doctor and PhD-student (JvA), a neurologist specialized in epilepsy (FL) and a medical doctor and ethicist (HvD). To achieve our aim, we applied VSD methodology, which consists of three elements: conceptual investigations, empirical investigations, and technical investigations [7,8].

*Conceptual investigations* identify relevant stakeholders and values that might influence the design of the device in question.

*Empirical investigations* provide information about the human context in which the device will be used.

*Technical investigations* are used to operationalize stakeholder values in terms of the technical features of the device.

### Empirical data collection

To ensure sufficient input from potential end-users, not only the members of the TeleEpilepsy consortium (1 nurse specialized in epilepsy care, 1 representative of commercial party, 4 engineers and 7 medical doctors specialized in epilepsy care) were consulted, but also a panel of 3 professional (caregivers in a residential care setting) and 10 informal caregivers (parents of children and adults with severe epilepsy) was formed. People with epilepsy were not involved in the panel. The large majority of people with epilepsy who will benefit from seizure detection are either young children or people with intellectual disability in addition to epilepsy. Direct consultation of these stakeholders on their values regarding the seizure detector was extremely difficult. Therefore, we obtained information about relevant aspects of the detector through caregivers.

One of the researchers (GT) recorded the values or priorities voiced by the members during consortium meetings and telephone conferences. She also analyzed all e-mail communication within the TeleEpilepsy consortium for statements about values or criteria for a ‘good’ system. This information was used to identify stakeholders and main values of interest. The possible benefits and risks of the device to potential stakeholders were defined for each value of interest. The main values identified were presented at a stakeholder meeting of the TeleEpilepsy consortium and a panel meeting, after which all stakeholders completed a survey (S1 Survey) on their interpretation, views, and comments about each of the main values. They were also asked to add information that they considered relevant and to rate the values in order of importance from 1 (least important) to 5 (most important).

Lastly, design choices were formulated, making use of the information about stakeholder risks and benefits, and reflecting main recurring themes observed by GT and JvA during stakeholder meetings. These formulations aimed at providing all stakeholders with a better understanding of the consequences of certain choices in the design process.

### Ethics statement

According to Dutch law, ethical approval by an institutional review board to conduct the questionnaire study was not required (CCMO Manual for the review of medical research involving human subjects,2002). Members of the panel of informal and professional caregivers were solicited through the Dutch epilepsy foundation. Prior to their participation, all participants were informed about the purpose of the study through an oral presentation (GT and JvA) and were made aware that the results would be used for publication. Informed consent was given verbally and confirmed by returning the questionnaire.

## Results

### Stakeholders

**Fig 1:**
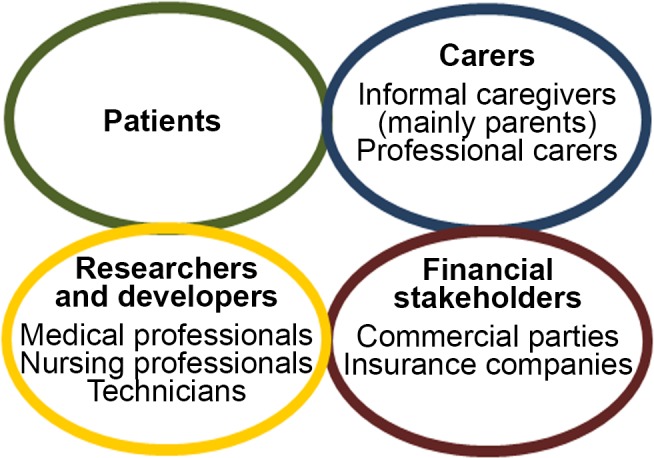
An overview of identified stakeholders.

We identified eight individuals or organizations interacting with the seizure detector, that would either be involved in the functioning or output of the device (such as medical and nursing staff or informal caregivers) or be affected by its use (patients) (Fig 1).

### Values, risks and benefits

In their work on VSD, Friedman and Kahn propose twelve “values of ethical import”: *human welfare*, *ownership and property*, *privacy*, *freedom from bias*, *universal usability*, *trust*, *autonomy*, *informed consent*, *accountability*, *courtesy*, *identity*, *calmness*, and *environmental sustainability*. [9] These values have ‘ethical status’ according to Friedman and Kahn. However, they are derived from the context of human-computer interaction, which may differ from the health care setting. Therefore, we used the 12 values as a heuristic starting point to identify relevant values for the development of a seizure detector. Another source of information on relevant values was the result of the empirical investigation into stakeholder communication. We compared both lists and in the end, our analysis of stakeholder communications identified five main values. We adopted Trust and Autonomy from Friedman and Kahn’s list and added Health, Reliability and Accessibility from the stakeholder communications. A more elaborate description of these values follows at the end of this section.

In a round of empirical investigation, stakeholders reported their interpretation and prioritization of these values. All 13 participants in the consortium meeting completed the survey during the meeting, whereas only 8 of the 13 panel participants did (5 participants needed more time to complete the survey and did not return the survey). In total, 21 surveys were analyzed from the following stakeholders: 5 informal caregivers, 3 professional caregivers, 1 nurse specialized in epilepsy care, 1 representative of commercial party, 4 technicians, and 7 medical professionals (neurologists). People with epilepsy were not included in this survey. Based on these reports, we divided the values in 11 subcategories. In addition, we operationalized the values into possible benefits and risks of the introduction of the seizure detector to these values. The values together with the associated risks and benefits should be used to guide design choices: these choices ideally maximize the identified benefits and minimize anticipated risks. In Table 1, a summary of the values, subcategories, benefits and risks is presented. In the following, we illustrate each (sub-)category.

**Table 1:** Values and their interpretations for the seizure detector.

Value	Subcategory	Benefit	Risk
**Health**	Patient safety	Detection of seizures	Physical harm caused by system (mis-)use
	Optimal care	Timely caregiving	Lack of availability of trained caregivers
	Optimal care	Objective information on seizures	Burden of new knowledge on seizures
	Optimal care	Respond to patient behaviour	-
	Quality of life	Improved sleep patients	Potential burden of sensors
	Quality of life	Improved sleep caregivers	-
**Trust**	Trust	Trust and relaxation for patients	‘Human factor’: device part of ‘care system’
	Trust	Trust and relaxation for caregivers	-
	Trust	Evidence based seizure detection	-
	Trust	Response to major seizures	-
	Trust	Response to ‘risk averse’ society	-
**Accessibility**	Usability	Personalized telecare	Lack of availability of trained caregivers
	Usability	Plug and play	Insufficient health capacities of end-users
	Usability	Zero maintenance	Technical problems
	Usability	Technical support	-
	Availability	Easy access to data on seizures	Potential for breach of privacy
	Availability	-	Costs
	Availability	-	Acceptance of prescribers and insurers
**Reliability**	Accuracy	Low false positives/false negatives	No 100% reliability possible
	Accuracy	Scientific validity	What is a clinically relevant seizure?
	Accuracy	Short response time	Over-monitoring may lead to alarm-fatigue
	Accuracy	Detection of multiple seizure types	-
	Technical	Sustainability	Technical failure
	Technical	Up-to-date	Interference of/with other systems
	Technical	Technical support available	-
	Technical	Remote control	-
**Autonomy**	System	Support patient autonomy	Diminished level of care
	System	More normal family life	-
	Privacy	-	Misuse of data and video imaging
	Privacy	-	Restriction of freedom
	Responsibility	Better circumstances for caregiver	Unclear division of responsibility
	Responsibility	-	Potential burden of responsibility

The five main values are shown with subcategories. Risks and benefits of the introduction of the seizure detector regarding each subcategory are summarized in this table.

#### 1. Health

Health is linked to the physical and mental functioning of people with epilepsy and caregivers. The subcategories of Health are patient safety, optimal care, and the quality of life of people with epilepsy and caregivers. The potential benefits that are ideally maximized in the development of the seizure detector are firstly the detection of seizures. In turn this should facilitate timely caregiving in case of a major seizure and improved sleep and relaxation of caregivers and people with epilepsy due to the alarm function of the device (an aspect of quality of life). Besides caregiving at the time of seizures, improved insight in nocturnal seizure frequency can help optimize the medical treatment of the epilepsy. Finally, caregivers pointed out that knowledge about the seizures in the night could help them decide about the best way to deal with fluctuating behavior during the day: if for example fatigue or resistance can be traced back to disease activity, a different pedagogical approach is needed compared to situations in which other causes for behavioral issues are more likely. Possible risks are the burden of sensors for the people wearing the device. In addition, information about nocturnal seizure frequency may not always serve the user’s interests. Potential users of the detector are adults and children with severe intractable epilepsy, with little options for further treatment. Knowledge of a larger burden of seizures without the possibility to treat these seizures may have a negative effect on their (mental) wellbeing.

#### 2. Reliability

This value refers directly to expectations of device performance. It is a traditional technical value which entails the extent to which the device accurately does what it is supposed to do, in this case to detect epileptic seizures. This in turn depends on the scientific validity of the device, and its sensitivity and specificity. Thus, a low rate of false positive and false negative alarms and a short response time are important benefits. On the side of the risks, it is important to note that 100% reliability is never possible. Too much emphasis on avoiding false negatives may lead to over-monitoring of seizures and subsequently to alarm fatigue. Determining which seizures are clinically relevant and need a response from a caregiver is also a challenge due to the large inter-individual variability. A second aspect of reliability is the technical functionality, which needs to be up-to-date, sustainable and when necessary supported by technical experts.

#### 3. Trust

Trust is a value which refers to interactions between people and their experiences of extending good will to one another and of being vulnerable. In the case of the seizure detector, users, for example, need to trust that the device is designed with their interests as the primary focus. This is especially important because of the vulnerability of the people with epilepsy and their caregivers. Moreover, the detector is an evidence based seizure detection system, which enhances its potential to support a sense of trust and relaxation for its users. On the other hand, the direct communication between designers, users and potential other caregivers can be limited when using complex technological devices. This may negatively affect the trust between stakeholders. In order to diminish the risk of low trust in the detector, it needs to be embedded in an environment in which the ‘human factor’ is preserved.

#### 4. Accessibility

*This value* concerns the ease of acquiring and using the device. The device is accessible if it is both usable and available. Benefits, like being easy to operate (plug and play) and requiring zero maintenance, enhance usability. Technical problems and lack of knowledge of end-users and limited availability of trained caregivers are risks to usability. With regard to availability, the detector can disclose much more data on seizures compared to currently available devices. Access to this data is considered a benefit by the stakeholders. On the other hand, easy access to personal data holds the risk of intrusion of the privacy of the users. When designing the device, this risk should be taken into account. Two other aspects that potentially limit the detector’s availability are its costs and the willingness of physicians and insurers to prescribe and reimburse the detector.

#### 5. Autonomy

Autonomy roughly refers to the extent to which an individual can live his or her life the way he or she wishes to do. A detection device can support autonomy, for example by allowing parents and children to sleep in their own bedrooms. The detector can give caregivers an increased sense of control, acting as an active coping tool. Also autonomy of people with epilepsy can be increased when there is a possibility of life outside a care facility due to the use of the detector in a home situation. On the other hand, constant monitoring of physical information (images, sound, heart rate, and movement) can interfere with autonomy, by restricting the privacy and the freedom of the person who is being observed. In addition, it may be unclear if and how responsibilities of caregivers change after the device is introduced. Examples of questions that may rise are: What is the responsibility of caregivers with regard to the adequate functioning of the detector? Will caregivers lower the number of care interventions or change their way of giving care to the person with epilepsy? This uncertainty about the new division of responsibility and the burdens associated with it, is a risk that is relevant in the process of design of the seizure detector.

### Priority of values

**Fig 2:**
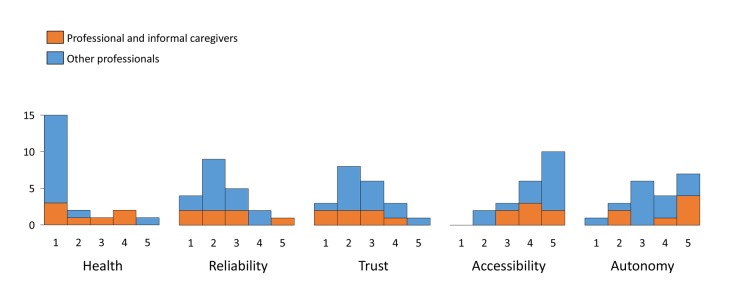
Histograms of priority given by surveyed stakeholders to the 5 identified main values. 1 is high priority, 5 is low priority.

At the end of the questionnaire, the respondents were asked to prioritize the five values. A histogram of priorities given to each value by the caregivers and by the other professionals from the tele-epilepsy consortium is presented in figure 2 (Fig 2). No clear individual or group outliers were identified. The value autonomy was not highly prioritized, which was especially prominent in the caregivers included in the survey.

### Operationalization of values in design choices

Mapping risks and benefits to the values that were identified was an important step to gain insight in the effects of certain features of the detector. In VSD, the next step is to operationalize the stakeholder values in terms of the technical features of the device. Based on the five main values and corresponding benefits and risks (Table 1), four design choices were formulated. These choices reflect main themes mentioned in stakeholder meetings, observed by GT and JvA. Each design choice has two extremes with corresponding risks and benefits. The risks and benefits of making the design choice give the developers an indication of the consequences of a design choice. It shows the developers which risks need to be addressed and which advantages or benefits can be maximized.

### Design choice 1: Level of detection of epileptic seizures

Design choice 1, the *level of detection of epileptic seizures*, reflects the balance between risks and benefits of a highly personalized device which is accurate for specific patients and a more general device only detecting major seizures. A general device is readily available and easy to use for many patients, but gives less accurate information. Only major seizures can be detected. A highly personalized system is more accurate and can also detect smaller seizures. This information can be useful in deciding on treatment options or adjusting medication, thereby facilitating more optimal care. On the other hand, a more personalized system is not as readily available as it requires an extra investment to adjust the settings of a device to a specific patient. Also, highly accurate seizure detection carries the risk of a potential burden of the knowledge of a higher seizure frequency than expected, without proper treatment options to reduce the seizure frequency. Another risk of personalized detection including detection of smaller seizures can be overmonitoring leading to alarm fatigue. When alarms lose urgency due to alarming for minor seizures, the response to actually urgent alarms can decrease (alarm fatigue), which can lead to potentially dangerous situations.

### Design choice 2: Quality of life and system usability

In choice 2, *quality of life and system usability*, are important. The choice involves the decision to either use remote sensors, such as video or radar technology, or wearable sensors. While a remote sensor disturbs the patient’s life less, and is easier to use as no application is necessary, it is less accurate than a wearable sensor. A wearable sensor more accurately measures relevant physiological signs such as movement and heart rate, but is more sensitive to technical failure or removal by the person wearing it.

### Design choice 3: Autonomy in the context of care and control

Design choice 3, *autonomy in the context of care and control*, concerns the operation of the detector, which can range from operation by informal caregivers to operation by trained professionals. A system operated by trained professionals is convenient for the caregiver as it comes with zero maintenance. It is also highly trusted, as professional caregivers are taking responsibility for seizure detection and care. Risks of a professionally operated system are high expenses and limited availability of professionals and a high invasion of privacy of the subject with epilepsy. A system operated by informal caregivers gives caregivers more autonomy, as they are in control of the detection system and care. It also facilitates timely caregiving, as an informal caregiver is usually already near the person with epilepsy. On the other hand it puts a high burden of responsibility on the informal caregiver: failing to attend to a detected seizure leading to harm to the person with epilepsy can be highly burdensome. If an informal caregiver does not have the capacities to provide proper care after a seizure, the device can overburden the caregiver. A final risk of a caregiver-operated device is the possibility of a lack of technical support in case of technical failure of the device.

### Design choice 4: Evidence-based versus trusted system

The fourth design choice, *evidence-based versus trusted system*, reflects an issue that was frequently raised by caregivers and medical professionals, namely, the question of seizure detection versus vital signs monitoring. Seizure detectors do not necessarily monitor vital signs. Parents of children with specific epilepsy syndromes that are accompanied by breathing disorders would like a device that also alerts them to apnea and a low oxygen saturation. In this case, the scientists developing the detector did not find evidence in the literature that monitoring vital signs influenced or improved seizure detection. The views of these important stakeholders did not completely overlap and both approaches come with certain risks and benefits such as scientific validity on the one hand and feeling of trust and diminished anxiety on the other. An evidence based system is of course more scientifically valid but the ‘human factor’ needs to be taken into account: when solely relying on evidence based sensors the caregiver may not trust the system and therefore will not use it. A trusted system with the extra, non-evidence based sensor could lead to better caregiving and quality of life of caregivers, as the caregivers will rely on the alarms and feel more relaxed. A disadvantage of this trusted system can be the potential burden of extra sensors, which are not strictly necessary for seizure detection. Also a system can never by fully trusted, as 100% reliability is not possible in practice. This means caregivers still need to accept the possibility of something going wrong, and find a way to come to terms with this situation. Striving for a fully reliable and trusted system is not a realistic aim and comes with the risk of continuous development without ever finishing and producing a detection device.

## Discussion

The main findings from our parallel ethics study are the identified values and their operationalization in terms of choices in the design of a seizure detector. In addition, our parallel ethics study provides experience and lessons for value sensitive design (VSD) in medical device development.

With regard to the values relevant for the design, we found that Health was the value with the highest priority among stakeholders, followed by Reliability and Trust. The seizure detector cannot influence someone’s health directly. Nonetheless, its indirect contribution to someone’s health may be of importance, if sufficient reliability is achieved. A reliable seizure detector facilitates timely caregiving in case of seizures. In a recent study on risk factors for Sudden Unexpected Death in Epilepsy (SUDEP), ‘no surveillance at night’ was identified as a moderate risk factor [10]. In addition, more knowledge on seizures may enable physicians and caregivers to optimize pharmaceutical and behavioral treatment. With regards to trust, we found that a trusted system for seizure detection is a true unmet need. The reliability of the system is an important value, which is connected to trust, but does not overlap the value of trust completely. To build and support trust, the perspective of technical experts (with their focus on evidence based device development) needs to be integrated in the experience-based perspective of users. For example, many caregivers at home used oxygen saturation meters to monitor at night. The caregivers’ trust in this type of monitoring cannot simply be overruled by scientific arguments generally used by technicians. In a value-based design process, it becomes clear that trust requires that relationships between stakeholders are established, in which relevant experiences and proof of willingness to benefit to patient are exchanged. Finally, we found that issues related to autonomy, such as the desire to promote normal (family-)life, an increased sense of self-control and issues of privacy were mentioned by stakeholders.

There is no consensus regarding the matter of which moral values should be taken into account in health care technology development. In the literature a variety of values relevant to design is reported. For example, values derived from a care ethics perspective (attentiveness, responsibility, competence and reciprocity) [11] or from well-established ethical principles of health care (autonomy, beneficence, non-maleficence and justice) [12]. In absence of clear guidance for identifying relevant values, we used Friedman’s list of 12 values as starting point. [9]. Our analysis of stakeholder communication was however decisive for the choice of values taken up in this study.

We were surprised to see Autonomy end up as lowest-priority value. In health care ethics, autonomy is generally considered a core value [13]. The discrepancy with our findings may be caused by the fact that in our case, there was no direct representation of the patient as stakeholder. The detector is intended for use with young people or people with intellectual disabilities who are generally unable to speak for themselves. Nonetheless, patient autonomy is relevant, even if patients have diminished capacities for autonomy. To shape autonomy in their lives, vulnerable groups like children and intellectually disabled people are largely dependent on their direct environment. The extent of acknowledgment of the value of autonomy by caregivers thus likely influences the possibilities for autonomy of patients dependent on them.

The design choices are based on discussions during meetings of the TeleEpilepsy consortium. In our approach, structured attention for values and their role in design was secured from the early beginning of the design process. The identified values, risks and benefits were attributed to design choices, yielding a very practical insight in possible consequences of certain design choices. This may be an important factor for success.

A limitation of the study is the small sample size of the surveyed stakeholders, and the unequal distribution of respondents over several stakeholder groups. The aim of the qualitative empirical inquiry was, however, to obtain the views on values of the individuals who were actually *involved* in designing the seizure detector. Therefore, purposeful sampling was impossible. On the other hand, we managed to include all involved stakeholders in the survey. A second weakness is the lack of direct representation of the patient as stakeholder. In our case they were represented by their caregivers. The limited autonomy of patients and their representation by caregivers is problematic in relation to our aim in two ways. First, patients and caregivers may have different perspectives on choices in the design. In an extensive survey of wearable sensor systems by Pantelopoulos & Bourbakis [14], there was discrepancy between users (patients), physicians, and producers in the evaluation of the importance of specific features of a sensor system. The three parties differed in their opinions about the relevance of features such as cost, comfort, and esthetics. Second, the fact that autonomy was not highly prioritized, may have been a result of the fact that patients were not autonomous. However, the stakeholders (including caregivers as patient representatives) may also have overlooked possibilities to stimulate patient autonomy. It is important to realize that in case of medical devices, many stakeholders are not truly independent, in the sense that they are dependent on their caregivers, and patients and caregivers are dependent on medical professionals, which makes interpretation of their values difficult as they may be influenced by information from other stakeholders who have other priorities.

In conclusion, we believe that incorporating operationalized values with specific risks and benefits into design choices, facilitates the design of a seizure detector according to stakeholder values. The design choices will be used to inform decision making on the further development of the seizure detector. However, the underrepresentation of the patient as stakeholder needs to be addressed in a separate way. This limits the comprehensiveness of our analysis of values, which is a serious shortcoming. Nonetheless, the insights that resulted from the identification and analysis of values seem to provide a workable basis for our aim to further develop the seizure detector in a value-sensitive manner.

## Supporting Information

S1 Survey Survey on interpretation and prioritization of values. (DOC)
