# Supplementary material for: What Makes a Good Home-Based Nocturnal Seizure Detector? A Value Sensitive Design
Source: PLoS One. 2015 Apr 13;10(4):e0121446. doi: 10.1371/journal.pone.0121446 (PMC4395301; doi:10.1371/journal.pone.0121446)
Supplement: S1 Survey — (DOC) [file pone.0121446.s001.doc]

**REFLECTION ON VALUES REGARDING DESIGN OF A HOME-BASED SEIZURE DETECTION SYSTEM FOR PEOPLE WITH EPILEPSY**

Name:

Background:

**To which group of stakeholders do you consider yourself to belong?**

0 Patient

0 Parent/informal caregiver of a person with epilepsy

0 Professional caregiver of a person with epilepsy

0 Engineer

0 Medical professional (neurologist or other physician)

0 Nurse

0 Representative of a commercial party 0 Representative of an insurance company

**Do you feel a stakeholder is missing in the list above?**

0 No

0 Yes, being _____________________________________________________________________

**ADDITIONS TO INTERPRETATION OF VALUES**

**Value 1 of 5**

**HEALTH**

The degree in which the detection device influences health of users of the device

**0 PATIENT SAFETY**

0 Providing care immediately after a seizure occurred

0 Risk of harm due to direct contact with the detection device

0 ______________________________________________________________________________________

0 ______________________________________________________________________________________

0 ______________________________________________________________________________________

0 ______________________________________________________________________________________

**0 OPTIMIZING CARE**

0 Information on nocturnal seizures facilitates more tailored treatment and therefore positively influences the health of the patient

0 ______________________________________________________________________________________

0 ______________________________________________________________________________________

**0 OTHER INTERPRETATIONS:**

0 ______________________________________________________________________________________

0 ______________________________________________________________________________________

0 ______________________________________________________________________________________

**VALUE 2 OF 5**

**TRUST**

The degree in which the system influences trust of users

0 Patient is more at ease

0 The burden of care is lower

**0 OTHER INTERPRETATIONS**

0 ______________________________________________________________________________________

0 ______________________________________________________________________________________

**VALUE 3 OF 5**

**ACCESSIBILITY**

The extent to which the system is usable and accessible to users

**0 USABILITY**

0 Degree of complexity

0 Possibility of tailoring to individual patient

0 Possibility of tailoring to different aims (i.e. alarming or diagnostic purpose)

0 ______________________________________________________________________________________

0 ______________________________________________________________________________________

**0 AVAILABILITY**

0 Number of patients for whom the device is usable

0 Cost

**0 OTHER INTERPRETATIONS**

0 ______________________________________________________________________________________ 0 ______________________________________________________________________________________

0 ______________________________________________________________________________________

**VALUE 4 OF 5**

**RELIABILITY**

Degree to which the system does what it is supposed to do

**0 ACCURACY**

0 Limited number of false alarms

0 Limited number of missed seizures

0 Scientific validity

0 ______________________________________________________________________________________

0 ______________________________________________________________________________________

**0 OPTIMAL TECHNICAL FUNCTIONING**

0 Device has few technical failures

0 ______________________________________________________________________________________

0 ______________________________________________________________________________________

**0 OTHER INTERPRETATIONS**

0 ______________________________________________________________________________________ 0 ______________________________________________________________________________________

**VALUE 5 OF 5**

**AUTONOMY**

The extent to which the system effects the ability and right of an individual to live his or her life the way he or she wishes to do

**0 SYSTEM CAPABILITY**

0 Facilitation of autonomous development of a child with epilepsy

0 Facilitation of normal family life

0 ______________________________________________________________________________________ 0 ______________________________________________________________________________________

**0 CONTROL**

0 The user is in control of the system

0 ______________________________________________________________________________________

0 ______________________________________________________________________________________

**0 PRIVACY**

0 Careful management of privacy-sensitive data

0 ______________________________________________________________________________________

0 ______________________________________________________________________________________

**0 RESPONSIBILITY**

0 Clear distribution of responsibility for the well-being of the patient

0 ______________________________________________________________________________________

0 ______________________________________________________________________________________

0 ______________________________________________________________________________________

**0 OTHER INTERPRETATIONS**

0 ______________________________________________________________________________________

0 ______________________________________________________________________________________

**PRIORITIES AND MISSING VALUES**

Can you prioritise the values stated below?

1 = most important considering the detection device

5 = least important considering the detection device

__ Health

__ Trust

__ Accessibility

__ Reliability

__ Autonomy

Are important values missing?

0 No

0 Yes, being __________________________________________________________
